# The Semantic Similarity Effect on Short-Term Memory: Null Effects of Affectively Defined Semantic Similarity

**DOI:** 10.5334/joc.349

**Published:** 2024-02-12

**Authors:** Sho Ishiguro, Satoru Saito

**Affiliations:** 1 Kyoto University, Japan

**Keywords:** semantic similarity, semantic relatedness, short-term memory, semantic maintenance

## Abstract

Studies on short-term memory have repeatedly demonstrated the beneficial effect of semantic similarity. Although the effect seems robust, the aspects of semantics targeted by these studies (e.g., categorical structure, associative relationship, or dimension of meaning) should be clarified. A recent meta-regression study inspired by Osgood’s view, which highlights affective dimensions in semantics, introduced a novel index for quantifying semantic similarity using affective values. Building on the results of the meta-regression of past studies’ data with that index, this study predicts that semantic similarity is deleterious to short-term memory if it is manipulated by affective dimensions, after controlling for other confounding factors. This prediction was directly tested. The experimental results of the immediate serial recall task (Study 1) and immediate serial reconstruction of order task (Study 2) indicated null effects of semantic similarity by affective dimensions and thus falsified the prediction. These results suggest that semantic similarity based on affective dimensions is negligible.

Short-term memory studies have repeatedly demonstrated that lists of semantically similar or related words lead to better serial recall performance than lists of semantically dissimilar or unrelated words (Kowialiewski et al., 2022; Neale & Tehan, 2007; Neath et al., 2022; Poirier & Saint-Aubin, 1995; Saint-Aubin & Poirier, 1999a; Tse, 2009; Tse et al., 2011; for akin effects on working memory, see Kowialiewski & Majerus, 2020; Rosselet-Jordan et al., 2022). This memory advantage is called *semantic similarity effect* (or *semantic relatedness effect*).^1^ Although the effect is robust, an important question is what facets of semantics have been targeted under the label of ‘semantic similarity’ given its complexity and multifaceted nature (Figure 1).

**Figure 1:**
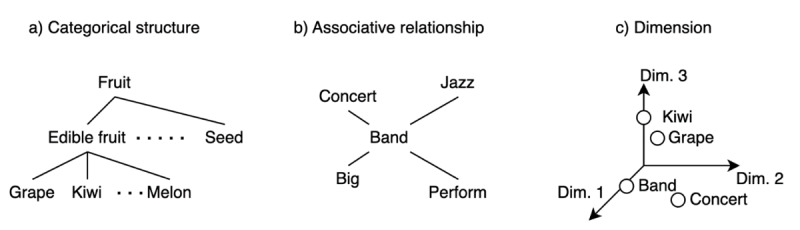
The schematic illustrations of the three facets of semantics. *Note*. **(a)** Categorical structure: Hierarchical categories are often assumed in semantics. **(b)** Associative relationship: Networks of associations would represent semantics. An association does not necessarily correspond to a category (e.g., the association between ‘big’ and ‘band’ is not based on category but rather on their contingency). **(c)** Dimensions: The meaning of a word is expressed as a vector of its values. In this figure, the spatial representation of meaning is depicted as a point in a multidimensional space.

In some studies on the semantic similarity effect (e.g., Poirier & Saint-Aubin, 1995), similar words were hyponyms (e.g., ‘grape, kiwi, melon’) of a hypernym (e.g., ‘edible fruit’), which relates to the *categorical structure* of semantics (e.g., hyponymy/hypernymy in WordNet, Miller, 1995; Miller et al., 1990). These studies have typically attributed the semantic similarity effect to a cue-dependent retrieval process (Neale & Tehan, 2007; Poirier & Saint-Aubin, 1995; Saint-Aubin et al., 2005; Saint-Aubin & Poirier, 1999a). For example, when participants are presented with ‘grape, kiwi, melon,’ they are assumed to generate and use ‘(edible) fruit’ as a retrieval cue that aids recall of these words. These studies typically use common and well-learned categories such as ‘edible fruit,’ ‘sports,’ and ‘musical instruments’ to create similar lists (e.g., Poirier & Saint-Aubin, 1995). The assumption of a cue-dependent retrieval process seems reasonable for studies defining semantic similarity using common and well-learned categories, as even uncommon or ad-hoc categories can affect memory performance (Barsalou, 1983; Gardiner et al., 1972; Saunders & MacLeod, 2006).

In other studies, similar words were thematically related (e.g., ‘band, concert, jazz’; e.g., Tse, 2009), which refers to *associative relationship* in semantics (De Deyne et al., 2019; Deese, 1962; Nelson et al., 2004). Consequently, semantic similarity effect can be explained by associative link-based processes (Kowialiewski & Majerus, 2020; Tse, 2009); for example, encoding/retrieving ‘band’ boosts the activation of representations of ‘concert’ and ‘jazz,’ which facilitates recall of ‘concert’ and ‘jazz.’ From a theoretical point of view, the spreading activation theory supposes associations: Collins and Loftus (1975) describe concepts connected to ‘red’ in a model as ‘the concepts associated with “red”’ (p. 412). Therefore, when semantics is modelled in terms of association, associative link-based processes, such as spreading activation, are theoretically justifiable.

Short-term memory studies have thoroughly addressed the categorical structure and associative relationship of meaning, even with computational models (Botvinick & Plaut, 2006; Kowialiewski et al., 2021; Kowialiewski & Majerus, 2020), probably because the effects pertinent to these two facets of semantics are readily mapped onto cue-dependent retrieval and associative link-based memory processes. However, the distinction between categorical structure and associative relationship has often been overlooked. For example, the results based on the operational definition of categorical structure were attributed to the effects of either categorical structure or associative relationship (see Ishiguro & Saito, 2021).

Another facet of semantics seldom considered by studies on the semantic similarity effect is *dimension*, which is frequently noted outside short-term memory research. For instance, computational models building on corpus data such as Latent Semantic Analysis (LSA; Landauer & Dumais, 1997) and word2vec (Mikolov et al., 2013) express the meaning of a word as a vector of values on dimensions (for a review on computational models of semantic memory, see Kumar, 2021). Psychological studies based on human ratings or task performance have reduced dimensionality and interpreted the dimensions of meaning (Hebart et al., 2020; Henley, 1969; Osgood et al., 1957; Osgood & Suci, 1955; Rips et al., 1973; Tranel et al., 1997; VanArsdall & Blunt, 2022; see also Deese, 1962). Through factor analysis of the data using the semantic differential method, Osgood and colleagues observe that the major dimensions of semantics are *affective* and that semantic similarity can be seen as spatial proximity in an affectively defined semantic space (Osgood et al., 1957; Osgood & Suci, 1955). Affective dimensions emerged in the factor analysis, even though Osgood and colleagues did not exclusively or intentionally select affective scales to assess meanings; this suggests that affective dimensions cover a major part of semantics. Recent findings from studies relating computational models to human ratings have also shown that affective dimensions are evident even in representations of LSA (Bestgen & Vincze, 2012; Hollis & Westbury, 2016; Recchia & Louwerse, 2015). Taken together, affective information is likely to comprise a major part of —albeit not the whole—semantics (Hollis & Westbury, 2016; Ishiguro & Saito, 2021; Majerus & D’Argembeau, 2011). Some studies on short-term memory have regarded affective effect as a class of semantic effects and accumulated evidence for the affective effect on short-term memory (Landry et al., 2022; Majerus & D’Argembeau, 2011; Monnier & Syssau, 2008; Tse & Altarriba, 2022; but see also Bireta et al., 2021).

A dimension-based definition of similarity would be appropriate for examining semantic similarity because it can directly address this between individual items’ values rather than the category–item relation of categorical structure or item–item relation of associative relationship. Categorical structure captures the category–item relation well (e.g., ‘edible fruit’–‘grape’) but does not necessarily quantify item–item similarity (e.g., Is the ‘grape’–‘kiwi’ pair more similar than the ‘grape’–‘melon’ pair?). Although associative relationship reflects item–item relation, association is different from similarity. In Hill et al. (2015), participants rated synonym pairs as more similar but *less associated* than antonym pairs, which is a counter-example to the view that association is identical to similarity.

Ishiguro and Saito (2021) conducted a meta-analysis of previous studies using an immediate serial recall task targeting the semantic similarity effect. Their results confirmed an overall similarity advantage (*dz* = 0.90). However, the meta-regression results demonstrated that the strength of manipulation on similarity according to affective dimensions decreased this advantage (Figure 2); that is, the more strongly an experiment manipulates semantic similarity in terms of affective dimensions, the smaller the similarity advantage. Ishiguro and Saito interpreted that the effects of categorical structure and associative relationship led to an overall similarity advantage (i.e., confounding effects for dimension-based similarity), whereas semantic similarity had a detrimental effect on short-term memory. They also made an extrapolative prediction that similarity advantage could turn into *similarity disadvantage* if the manipulation on similarity is strong enough and other confounding effects are controlled for. The present study tested this hypothesis.

**Figure 2:**
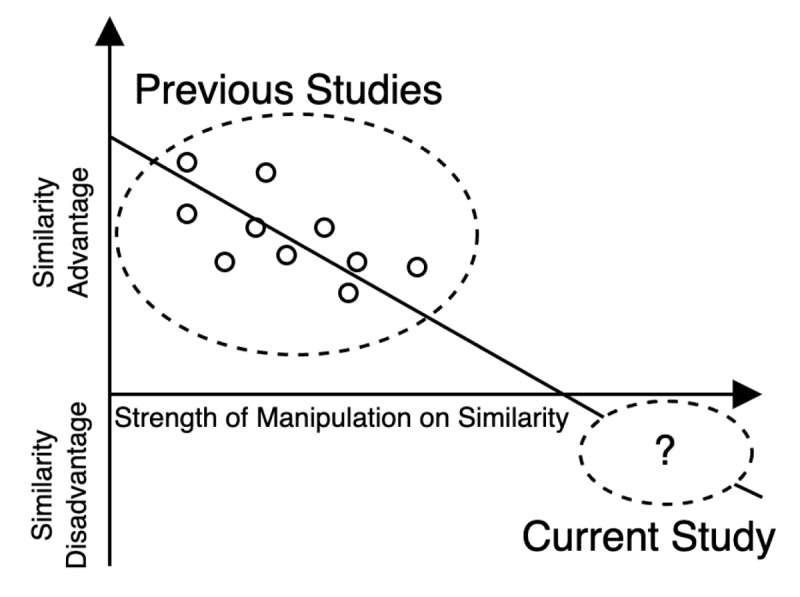
The schematic illustration of the relationship between similarity advantage and the strength of manipulation on similarity. *Note*. Each point represents each of the previous studies (i.e., effect size of similarity advantage and manipulation strength of a single experiment). Values are not accurate for presentation.

Recently, Kowialiewski et al. (2023) reported no credible detrimental effects of affectively defined semantic similarity as proposed by Ishiguro and Saito (2021). Although their analysis used the index proposed by Ishiguro and Saito (2021), their experiment manipulated the categorical structure and did not control for associative relationship (in some experiments, they manipulated phonological similarity based on rhyme categories). To the best of our knowledge, no studies have experimentally manipulated affectively defined semantic similarity while controlling for the factors of categorical structure and associative relationship. Thus, the current study aimed to manipulate affectively defined semantic similarity while controlling for the other factors.

Theoretically, numerous models of short-term memory posit that similarity leads to confusion or competition between item representations and is thus detrimental to memory (e.g., Botvinick & Plaut, 2006; Henson, 1998; Page & Norris, 1998; for a review, see Hurlstone et al., 2014). The assumption on similarity is evidenced by the detrimental effects of phonological and visual similarity (Avons & Mason, 1999; Baddeley et al., 1984; Conrad, 1964; Saito et al., 2008; but see also Kowialiewski et al., 2022); therefore, a disadvantage of semantic similarity is conceivable.

## Study 1

Study 1 conducted a within-participants design experiment with the immediate serial recall task and adopted correct-in-position as the primary scoring method to ensure consistency in the data targeted by the previous meta-regression study (Ishiguro & Saito, 2021).

### Method

#### Participants

One hundred participants were recruited online via Prolific. Data from one participant were not recorded and were substituted with additional participant data. Data from 100 participants (age: *M* = 25.09 years, *SD* = 3.04; gender: 47 women, 49 men, 4 other) were analyzed. The recruitment criteria were as follows: (a) their first language was English; (b) they resided in the US, UK, or Canada; (c) their nationality was US, UK, or Canada; (d) they were 20–31 years old; (e) they had no language-related disorders or cognitive impairments; (f) they had normal or corrected-to-normal vision; and (g) their approval rates in Prolific were equal to or over 90%. They were compensated with £4.50 for their participation (the task was completed within approximately 30 min).

#### Materials

We took steps for list construction to minimize the effects arising from categorical structure and/or associative relationship but to maximize the semantic similarity effect based on affective values. First, we applied *k*-means clustering to 600 two-syllable noun words used in a previous study (Ishiguro & Saito, 2020) by affective values (Warriner et al, 2013). In Warriner et al. (2013), participants rated how they felt while reading each word on 9-point scales; valence: 1 (happy) to 9 (unhappy); arousal: 1 (excited) to 9 (calm); and dominance: 1 (controlled) to 9 (in control). We retrieved the mean ratings for each word from the norms and used them as affective values. In the *k*-means clustering, we set *k* = 12 and obtained 12 clusters of words based on valence, arousal, and dominance values. Thus, each cluster contained words located closely to each other in the valence-arousal-dominance semantic space. Second, we applied hierarchical clustering to 12 centroids of clusters. Using a cluster dendrogram and visual inspection of the centroids’ spatial proximity, we allocated 12 clusters to either Set 1 or Set 2 (six clusters each), so that the centroids of clusters in a set were remote from each other. Dividing the 12 clusters into two sets facilitated the creation of dissimilar lists (see the later section on dissimilar list construction).

**Figure 3:**
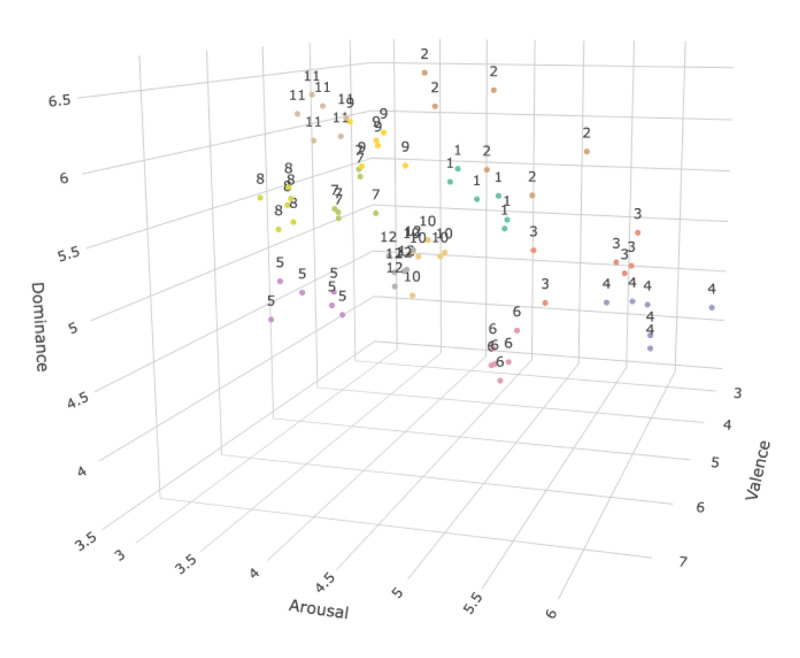
A plot of words of the 12 similar lists along the valence, arousal, and dominance dimensions. *Note*. Each number represents each similar list. For dissimilar list construction, 12 similar lists were divided into two sets. Set 1: similar lists 3, 4, 5, 9, 11, and 12. Set 2: similar lists 1, 2, 6, 7, 8, and 10. A dissimilar list was created by drawing one word from each similar list of a set (e.g., drawing a word from similar lists of Set 1). For an interactive plot, see a Jupyter notebook on OSF (https://osf.io/f4vb5).

Third, to construct a similar list, we selected six words for each cluster. We chose the word closest to the centroid and then entered the *N*th closest word when the resulting list met the criteria of categorical structure and associative relationship. Ensor et al. (2021) quantified categorical structure as *path length* in the WordNet database (Miller, 1995; Miller et al., 1990), which refers to the number of steps a word requires to reach another word in a categorical structure (for details, see Ensor et al., 2021). In their study, the mean path length between words in unrelated lists was 9.33. We adopted the value of 9.33 as a criterion: the mean path length values for our similar lists were all greater than 9.33; therefore, hyponyms hardly constitute a similar list. Associative relationship can be quantified using free association norms (De Deyne et al., 2019). These norms documented participants’ free responses to cues: for example, ‘yellow,’ ‘fruit,’ or ‘apple’ would be responses to the cue ‘banana,’ and thus, we can assume that ‘banana’ is associated with ‘yellow, ‘fruit,’ and ‘apple.’ Associative strength is the probability of a response to a given cue (e.g., if 20 out of 100 raters answered ‘yellow’ to ‘banana,’ the associative strength between ‘banana’ and ‘yellow’ was 0.20; Ishiguro & Saito, 2021). For our criterion, selected words were used as cue words in the norms (De Deyne et al., 2019) but were not cues or responses to any other words in a similar list (i.e., associative strengths are all zero). Therefore, the 12 similar lists of six words were similar in terms of dimensions (of affective values) but not similar in terms of categorical structure and/or associative relationship. Similar lists are presented in Figure 3. An example similar list is ‘complaint, glutton, dandruff, termite, despair, omen’ (see the points with the number 6 in Figure 3). Each word in this list has low valence, nearly medium arousal, and moderately low dominance values, indicating that the words are semantically similar in terms of affective dimensions. By contrast, an obvious categorical structure or associative relationship is lacking in the list.

Fourth, for the dissimilar list construction, we drew a word from six similar lists of a set and allocated the six selected words to a dissimilar list (i.e., recombination of words in similar lists). Thus, 72 words were used twice, once on a similar list and once on a dissimilar list, which would equate the effects of individual words’ properties (e.g., imageability) for the two types of lists. The centroids of six clusters of a set were remote from each other; thus, drawing a word from six similar lists based on six clusters resulted in a dissimilar list. As in the similar list construction, the criteria for categorical structure (9.33 mean path length) and associative relationship (0 associative strength) were adopted.

Words were sequentially evaluated from the second to *N*th closest words for similar list construction or were randomly sampled for dissimilar list construction until the resultant lists met the criteria. The resulting similar and dissimilar lists were matched for path length (for similar lists, *M* = 11.22, *SD* = 1.28; for dissimilar lists, *M* = 11.64, *SD* = 1.05; *t*(22) = 0.84, *p* = 0.41) and for association (all 0 associative strengths). The Jupyter notebooks used for list construction and the constructed lists are available at https://doi.org/10.17605/OSF.IO/VTPZK in the Open Science Framework (OSF).

Ishiguro and Saito (2021) proposed *Strength of Manipulation on Similarity* (SMS) to quantify the semantic similarity manipulation of an experiment. The SMS value is calculated based on the materials used in the experiment: it increases when dissimilar lists include words dispersed in the valence-arousal-dominance semantic space and/or similar lists include close words in that space. The SMS values of previous studies included in Ishiguro and Saito’s (2021) range from 0.11 to 0.46, whereas the value of the current study is 1.22. Therefore, the current study’s manipulation is assumed to be strong and beyond the range of previous studies (i.e., extrapolation). Ishiguro and Saito (2021) also reported the results of regression (p. 398). Setting association strength = 0, the regression equation for the current study would be


1 \[semantic\ similarity\ advantage\left({dz} \right) = -3.31 SMS + 1.71.\]

By plugging the current study’s SMS (1.22) to the equation, *dz* of –2.33 is predicted (i.e., the semantic similarity disadvantage).

#### Procedure

We conducted a web-based task. Participants who provided consent participated in four practice trials, the first block of 12 test trials, one filler trial, and the second block of 12 test trials. The presentation of list types (i.e., similar vs. dissimilar) was blocked and counterbalanced: 50 participants worked on similar lists in the first block and dissimilar lists in the second block (similar first group), while the other 50 worked in the reverse order (dissimilar first group). The presentation orders of test trials in a block and words in a trial were randomized for each participant, and a filler trial was inserted to avoid presenting the same words as those in the previous trial. The words used in practice and filler trials differed from those used in test trials. Practice, test, and filler trials were performed as follows.

A trial began with a fixation cross for 750 ms. After a 250-ms blank, six words were presented, with one word per second (750 ms on; 250 ms off). At the end of the trial, the participants were required to type the words individually. Each page asked about *N*th word (e.g., ‘Please type 1^st^ word’ on the first page). When they could not recall, they typed ‘skip.’

### Results

We report the results of frequentist and Bayesian analyses. For Bayesian analysis, we used *anovaBF* function of *BayesFactor* R package, version 0.9.12–4.4 (Morey et al., 2022) with default settings. We adopted a top-down analysis, comparing the full model with the full model dropping the target effect’s term, and reported Bayes factors correspond to main or interaction effects. Using Jeffreys’ classification scheme (Jeffreys, 1961, p. 432), we interpreted Bayes factors (BFs) as follows; not worth more than a bare mention: 1 < BF < 3.16; substantial: 3.16 < BF < 10; strong: 10 < BF < 31.62; very strong: 31.62 < BF < 100; and decisive: 100 < BF.

#### Correct-in-position

**Figure 4:**
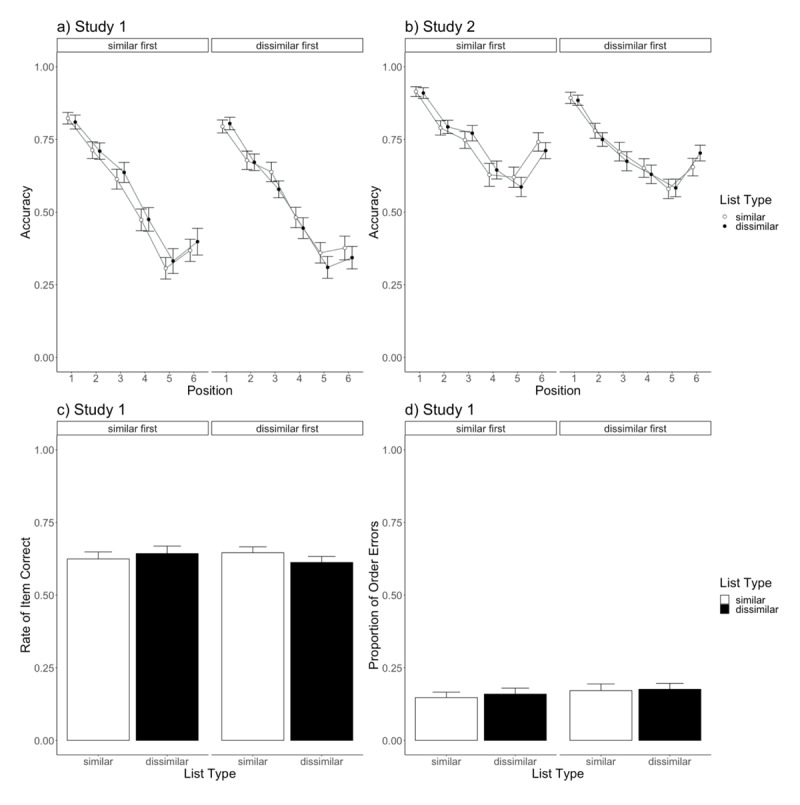
The results of Study 1 and Study 2. *Note*. The upper plots **(a)** and **(b)** represent correct-in-position scores in Study 1 (serial recall) and Study 2 (reconstruction of order), respectively. Accuracy refers to the rates of correct-in-position scores. Error bars represent standard errors calculated at each level combination (e.g., a standard error of the 50 participants’ scores of the similar first group at position 1 with dissimilar lists). The lower plots show the results of Study 1 by two scoring methods: **(c)** item correct scores and **(d)** proportion of order errors.

Correct-in-position scoring assigned scores to responses if these were recalled at their correct positions. Accuracy (rate) was calculated (Figure 4a).

We submitted correct-in-position data to an analysis of variance (ANOVA) with List Type (within-participants factor: similar vs. dissimilar) × Serial Position (within-participants factor: 1 – 6) × Presentation Order (between-participants factor: similar first vs. dissimilar first). It revealed the main effect of Serial Position, with *F*(5, 490) = 209.64, *p* < 0.001; η^2^*_G_* = 0.35; BF = 1.71 × 10^193^, but neither the main effects of List Type—*F*(1, 98) = 0.86, *p* = 0.36; η^2^*_G_* = 0.00; BF = 0.12 (for similar list, *M* = 0.55, *SD* = 0.29; for dissimilar list, *M* = 0.54, *SD* = 0.30)—nor Presentation Order—*F*(1, 98) = 0.16, *p* = 0.69; η^2^*_G_* = 0.00; BF = 0.30 (for similar first, *M* = 0.56, *SD* = 0.30; for dissimilar first, *M* = 0.54, *SD* = 0.29). None of the interaction effects reached statistical significance: List Type × Serial Position, *F*(5, 490) = 0.20, *p* = 0.96; η^2^*_G_* = 0.00; BF = 0.00; List Type × Presentation Order, *F*(1, 98) = 3.87, *p* = 0.05; η^2^*_G_* = 0.00; BF = 1.11; Serial Position × Presentation Order, *F*(5, 490) = 0.44, *p* = 0.82; η^2^*_G_* = 0.00; BF = 0.01; and List Type × Serial Position × Presentation Order, *F*(5, 490) = 1.50, *p* = 0.19; η^2^*_G_* = 0.00; BF = 0.03.

Our primary interest was in the effect of List Type but did not reach statistical significance. Furthermore, the effect size η^2^*_G_* was almost 0, and the BF favors the model without the List Type term (inverse BF = 8.44, which is substantial evidence for the null effect). Thus, the results suggest the null effect of semantic similarity defined by affective values, contrary to the prediction of Ishiguro and Saito (2021) (and the current study’s prediction).

#### Other scorings

We briefly report data by item correct and proportion of order errors scoring for completeness. For item correct, a response was scored as correct if it was a target word of that trial irrespective of its recalled position (i.e., free recall criteria). Proportion of order errors refers to the number of target words recalled at wrong positions divided by the number of recalled target words. This is a rate of order errors corrected with item correct. Given that order errors cannot be observed unless items are recalled and that better item memory would accidentally lead to more observations of order errors, such correction is needed. It is assumed that item correct mainly reflects item memory, while proportion of order errors reflects order memory (e.g., Saint-Aubin et al., 2005; Saint-Aubin & Poirier, 1999b, 1999a; Tse, 2009; Tse et al., 2011). Plots of item correct and order errors scores are shown at the bottom of Figure 4.

We submitted these data to an ANOVA with List Type (within-participants factor: similar vs. dissimilar) × Presentation Order (between-participants factor: similar first vs. dissimilar first). The results of item correct did not indicate the main effects of List Type—*F*(1, 98) = 0.77, *p* = 0.38; η^2^*_G_* = 0.00; BF = 0.20—or Presentation Order—*F*(1, 98) = 0.02, *p* = 0.88; η^2^*_G_* = 0.00; BF = 0.45 (Figure 4c). The interaction effect was significant, with *F*(1, 98) = 10.01, *p* = 0.002; η^2^*_G_* = 0.01; BF = 15.92, but it may simply reflect the effect of the repetitive presentation of words. The same words, although in different combinations and orders, were used for the two blocks, and item memory would be higher in the second block. The number of correct items for the second block (*M* = 0.64, *SD* = 0.16) was higher than that for the first block (*M* = 0.62, *SD* = 0.16), *t*(99) = 3.17, *p* = 0.002; *dz* = 0.32; BF = 11.63. The proportion of order errors showed no statistically significant effects for List Type—*F*(1, 98) = 0.73, *p* = 0.40; η^2^*_G_* = 0.00; BF = 0.21—Presentation Order—*F*(1, 98) = 0.53, *p* = 0.47; η^2^*_G_* = 0.01; BF = 0.46—or their interaction—*F*(1, 98) = 0.13, *p* = 0.72; η^2^*_G_* = 0.00; BF = 0.22 (Figure 4d). Regarding the main effect of List Type, the results of item correct and proportion of order errors scorings showed a null effect as in correct-in-position scoring.

### Discussion

The current experiment complemented previous studies that targeted the beneficial effects of categorical structure and associative relationship, by showing the absence of a beneficial effect of semantic similarity when controlling for categorical structure and associative relationship. Importantly, the effect size and Bayes factor indicate the null effect of semantic similarity defined by affective values on immediate serial recall. The results of Study 1 contradicted our prediction, although they may have been specific to the selected task. Next, another task was introduced (Study 2).

## Study 2

The previous meta-regression (Ishiguro & Saito, 2021) used data obtained from the immediate serial recall task, with which its prediction could be tested. However, given the null effect in Study 1, an attempt with another task was desirable. Study 2 thus replaced the immediate serial recall task with the immediate serial reconstruction of order task.

The immediate serial reconstruction of order task would be sensitive to the possible effect of semantic similarity because this task is likely to tap order memory (Saint-Aubin & Poirier, 1999b; Whiteman et al., 1994) and semantic similarity may affect order memory (Poirier et al., 2015; Saint-Aubin et al., 2005; but see also Neath et al., 2022).^2^ In fact, a classical study with the immediate serial reconstruction of order task reported a small but significant detrimental effect of semantic similarity (Baddeley, 1966).^3^

### Method

#### Participants

The experiment included 100 participants recruited via Prolific (age: *M* = 25.38, *SD* = 3.58; gender: 49 women, 50 men, 1 other). Recruitment criteria were the same as those in Study 1 except for an additional criterion of not having taken part in Study 1. The participants were compensated £3.00 for their participation. The participation fee was changed from that in Study 1 because a pilot study revealed that it took less time to click words in serial reconstruction of order (Study 2) than to type them in serial recall (Study 1). The task was completed in approximately 20 min.

#### Materials

The material in Study 1 was used in Study 2.

#### Procedure

**Figure 5:**
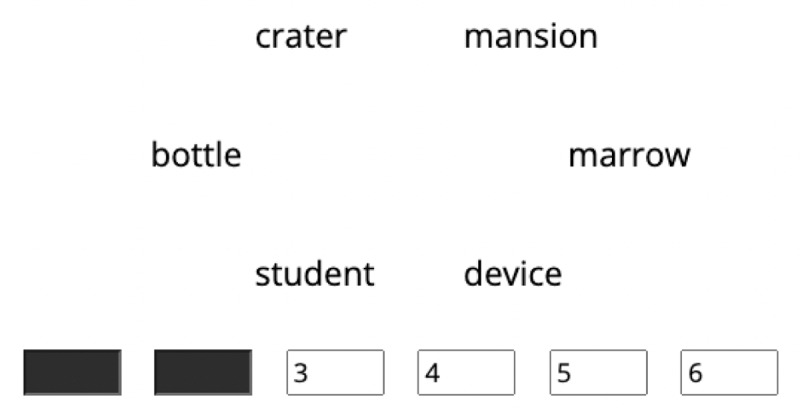
An example of test phase of the immediate serial reconstruction of order task. *Note*. This example depicts the moment when the third word is going to be selected. The first and second words have been selected, and thus, two boxes below are black.

Participants who gave their consent proceeded to the web-based task. The task had four practices, 12 tests (first block), one filler, and 12 test (second block), similar to Study 1. The procedure was identical to that of Study 1 until the test phase: it was changed from recall to reconstruction of order (Saito et al., 2021). At the end of a trial, six words were presented again on the screen (Figure 5). Each word appeared in one of six positions in a circle. The six positions were spatially fixed, but words were randomly allocated to these positions. Participants were asked to click the words in the order in which they were presented. Six boxes below displayed progress and turned black one by one (e.g., when the participant clicked the first word, the leftmost box with the number 1 turned black). In this task, the ‘skip’ option did not appear, and participants were allowed to click the same word(s) twice or more than twice.

### Results

Words clicked at their correct position were scored as correct (i.e., correct-in-position), and accuracy (rate) was calculated (Figure 4b, the upper right plot in Figure 4). ANOVA with List Type × Serial Position × Presentation Order revealed the main effect of Serial Position—*F*(5, 490) = 104.43, *p* < 0.001; η^2^*_G_* = 0.20; BF = 1.29 × 10^102^—and the three-way interaction effect of List Type × Serial Position × Presentation Order—*F*(5, 490) = 3.26, *p* = 0.01; η^2^*_G_* = 0.00; BF = 0.16. No other effects reached significance: the main effect of List Type, with *F*(1, 98) = 0.21, *p* = 0.65; η^2^*_G_* = 0.00; BF = 0.09 (for similar list, *M* = 0.73, *SD* = 0.23; for dissimilar list, *M* = 0.72, *SD* = 0.22); the main effect of Presentation Order, with *F*(1, 98) = 1.00, *p* = 0.32; η^2^*_G_* = 0.01; BF = 0.40); the interaction effect between List Type × Serial Position, with *F*(5, 490) = 0.37, *p* = 0.87; η^2^*_G_* = 0.00; BF = 0.00; the interaction effect between List Type × Presentation Order, with *F*(1, 98) = 0.01, *p* = 0.91; η^2^*_G_* = 0.00; BF = 0.10; or the interaction effect between Serial Position × Presentation Order, with *F*(5, 490) = 1.35, *p* = 0.24; η^2^*_G_* = 0.00; BF = 0.05.

### Discussion

The three-way interaction effect in frequentist analysis was statistically significant, but its effect size was almost 0. The Bayes factor favors the model dropping the three-way interaction term (inverse BF = 6.40). We suggest that this is a rather haphazard result; more importantly, the main effect of List Type was not statistically significant, and its effect size was negligible. Inverse BF (11.63) showed strong evidence for the null effect of List Type. The experiments with two tasks—the immediate serial recall task (Study 1) and the immediate serial reconstruction of order task (Study 2)—converged to show the null effect of semantic similarity defined by affective values. The results of Studies 1 and 2 clearly falsify the extrapolative prediction.

## General Discussion

The beneficial effect of semantic similarity on short-term memory has been documented (e.g., Neale & Tehan, 2007; Poirier & Saint-Aubin, 1995; Saint-Aubin & Poirier, 1999a; Tse, 2009; Tse et al., 2011). Nevertheless, this similarity advantage is elusive because the definition of semantic similarity varies across studies. As affective dimensions are major dimensions of semantics (Hollis & Westbury, 2016; Ishiguro & Saito, 2021; Majerus & D’Argembeau, 2011; Osgood et al., 1957), a definition based on affective values can guide our understanding of the semantic similarity effect. In a previous meta-regression study, Ishiguro and Saito (2021) proposed an index for semantic similarity based on affective values and calculated the index values for past studies using the immediate serial recall task. They found a negative relationship between the similarity advantage and the strength of manipulation of semantic similarity: if an experiment used materials that led to a strong manipulation of semantic similarity, the similarity advantage in that experiment declined. They further made an extrapolative prediction that the similarity advantage would turn into similarity disadvantage if the manipulation on similarity is strong enough. Study 1 tested this prediction but rejected it. The results of Study 1 did not show differences in immediate serial recall performance for similar versus dissimilar lists (i.e., List Type). The Bayes factor favors the null effect of List Type. Study 2 replaced the immediate serial recall task with an immediate serial reconstruction of order task, which again supports the null effect of List Type. The results of Studies 1 and 2 reject Ishiguro and Saito’s (2021) prediction.

Despite the null results, this study makes two major contributions to the literature. First, it aids in deconstructing ‘semantic similarity.’ As mentioned in the Introduction, the conceptual and operational definitions of semantic similarity are confused in the literature. Referring to models of semantics (e.g., De Deyne et al., 2019; Miller, 1995; Osgood et al., 1957), the current study clarified three definitions of semantic similarity of categorical structure, associative relationship, and dimension. It also introduced an experimental setting in which the effect of the semantic dimension was targeted but the effects of categorical structure and/or associative relationship were controlled. The null results for the semantic dimension in the current study complement those of previous studies indicating the facilitative effects of categorical structure and/or associative relationship. Second, testing an extrapolative prediction is valuable as extrapolation provides a prediction worth testing but does not guarantee its validity. For example, even if a linear relationship between age and short-term memory span is found in children, it cannot be applied to elderly people; therefore, an empirical investigation of the elderly population is necessary. Thus, the results of Studies 1 and 2 provide valuable evidence for testing and rejecting the extrapolative prediction proposed by Ishiguro and Saito (2021). Guided by this prediction, this study introduced a novel experimental setting in which semantic dimensions were selectively manipulated while controlling for the effects of categorical structure and associative relationship.

Although Ishiguro and Saito (2021) implied the possible detrimental effects of affectively defined semantic similarity, their evidence is based on past studies that did not control for the effects of categorical structure and/or associative relationship. In other words, affective dimensions may have a detrimental effect under the influence of categorical structure and/or associative relationship. The ideal experimental setting for focusing on the affective dimensions’ effect, proposed by Ishiguro and Saito (2021), should control for effects of categorical structure and/or associative relationship. The current study was such a setting. Since it showed null results, we tentatively conclude that the semantic similarity effect based on affective dimensions *by itself* is negligible. Given the complexity and multifaceted nature of semantics, we call for future studies examining each facet of semantics (e.g., categorical structure, associative relationship, and dimension) to grasp the whole picture of the semantic similarity effect.

## Data Accessibility Statements

Materials, anonymized data, and analysis scripts are available on the OSF (https://doi.org/10.17605/OSF.IO/VTPZK).

## Footnotes

‘Semantic similarity’ and ‘semantic relatedness’ are often used interchangeably. In this paper, we use the term ‘semantic similarity’ for consistency. To avoid jingle-jangle fallacies, the conceptual and operational definitions of ‘semantic similarity’ are of primary importance.

The results of proportion of order errors of Study 1, however, indicated the null effect of semantic similarity on order memory.

The definition of semantic similarity in that study is unclear, and the researcher may have selected words subjectively.
